# Association between changes in lumbar Modic changes and low back symptoms over a two-year period

**DOI:** 10.1186/s12891-015-0540-3

**Published:** 2015-04-22

**Authors:** Jyri Järvinen, Jaro Karppinen, Jaakko Niinimäki, Marianne Haapea, Mats Grönblad, Katariina Luoma, Eeva Rinne

**Affiliations:** Department of Diagnostic Radiology, Institute of Diagnostics, Oulu University Hospital, Oulu, Finland; Center for Life Course Epidemiology and Systems Medicine, Faculty of Medicine, University of Oulu, Oulu, Finland; Medical Research Center Oulu, University of Oulu and Oulu University Hospital, Oulu, Finland; Finnish Institute of Occupational Health, Health and Work Ability, and Disability Prevention Centre, Oulu, Finland; Department of Physical and Rehabilitation Medicine, Institute of Clinical Sciences, University of Helsinki, Helsinki, Finland; University of Helsinki and HUS Imaging Center, Helsinki University Central Hospital, Helsinki, Finland

**Keywords:** Modic changes, Prospective study, Low back pain, Change of symptoms

## Abstract

**Background:**

The association of Modic changes (MC) with low back pain (LBP) is unclear. The purpose of our study was to investigate the associations between the extent of Type 1 (M1) and Type 2 (M2) MC and low back symptoms over a two-year period.

**Methods:**

The subjects (*n* = 64, mean age 43.8 y; 55 [86%] women) were consecutive chronic LBP patients who had M1 or mixed M1/M2 on lumbar spine magnetic resonance imaging (MRI). Size and type of MC on sagittal lumbar MRI and clinical data regarding low back symptoms were recorded at baseline and two-year follow-up. The size (%) of each MC in relation to vertebral size was estimated from sagittal slices (midsagittal and left and right quarter), while proportions of M1 and M2 within the MC were evaluated from three separate slices covering the MC. The extent (%) of M1 and M2 was calculated as a product of the size of MC and the proportions of M1 and M2 within the MC, respectively. Changes in the extent of M1 and M2 were analysed for associations with changes in LBP intensity and the Oswestry disability index (ODI), using linear regression analysis.

**Results:**

At baseline, the mean LBP intensity was 6.5 and the mean ODI was 33%. During follow-up, LBP intensity increased in 15 patients and decreased in 41, while ODI increased in 19 patients and decreased in 44. In univariate analyses, change in the extent of M1 associated significantly positively with changes in LBP intensity and ODI (beta 0.26, *p* = 0.036 and beta 0.30, *p* = 0.017; respectively), whereas the change in the extent of M2 did not associate with changes in LBP intensity and ODI (beta -0.24, *p* = 0.054 and beta -0.13, *p* = 0.306; respectively). After adjustment for age, gender, and size of MC at baseline, change in the extent of M1 remained significantly positively associated with change in ODI (beta 0.53, *p* = 0.003).

**Conclusion:**

Change in the extent of M1 associated positively with changes in low back symptoms.

## Background

Modic changes (MC) are vertebral subchondral bone marrow changes that are visible in magnetic resonance imaging (MRI). They are strongly associated with degenerative disc disease [1]. MC, especially Type 1 MC (M1), have been correlated with low back pain (LBP) in both population-based and clinical samples [2-6].

Three main types of MC have been described. M1 shows decreased signal intensity on T1-weighted images (T1w) and increased signal intensity on T2-weighted images (T2w) [1]. M1 is thought to represent acute inflammatory changes in degenerative disc disease, on the basis of fibrovascular replacement in histopathological specimens of subchondral bone marrow [1,2]. It has been suggested that M1 may predict a fast-progressing and deforming type of disc degeneration [7]. M1 has also been linked to an inflammatory pain pattern in clinical contexts [8]. Type 2 MC (M2) shows increased signal intensity on both T1w and T2w, and it appears as yellow marrow replacement in histopathological specimens. M2 could represent a more stable phase of degenerative disc disease, but it does have the potential to convert to another type [9-13]. Type 3 MC (M3) shows decreased signal intensity on both T1w and T2w and is associated with extensive subchondral bone sclerosis on plain radiographs [1,14,15]. Mixed Modic types are thought to develop when one Modic type converts to another [16].

Only a few follow-up studies have evaluated the role of MC types and MC conversions in relation to low back symptoms [5,17]. The presence of M1 at both baseline and 14-month follow-up was found to be associated with poor outcome in patients with persistent LBP and MC [5]. Moreover, it has been suggested that as M1 converts to M2, pain intensity and perceived disability subside [18]. The aim of this study was to investigate associations between changes in the size and type of MC and low back symptoms over a two-year follow-up.

## Methods

### Study population

The study population was selected from consecutive LBP patients (*n* = 4380) with or without radicular symptoms who were referred initially for standard lumbar spine MRI to the Departments of Orthopaedics, Rheumatology or Physical and Rehabilitation Medicine at the region of Helsinki University Hospital during 2003–2007. Images from all patients examined by MRI were analysed monthly, and eligible patients were identified by an experienced radiologist. The inclusion criteria were chronic nonspecific LBP of at least three-month duration and lumbar M1 or mixed M1/M2 [18]. All included patients gave written informed consent to use their clinical data for study purposes. The study protocol was approved by the Ethics Committee of Helsinki and the Uudenmaa District University Hospitals.

The exclusion criteria were age ≥ 65 y; specific back disease, such as fracture, neoplasia, infectious, or rheumatic spine disease; spondylolisthesis (≥4 mm); spinal stenosis; disc extrusion; any other finding with even the slightest neural compression; minor spine operation, such as herniated disc surgery within the past six months; and major spine operation, such as fusion or disc prosthesis at any time. Annular tears, bulging of the disc, and facet joint degeneration were not exclusion criteria, since these changes are often found in association with disc degeneration and also with MC. When there was uncertainty about the etiology of signal abnormalities, we checked laboratory results and other clinical findings to exclude specific causes (e.g., infectious or rheumatic spinal disease).

Within 1–3 weeks of identification, eligible patients were contacted by telephone to complete questionnaires to describe average LBP intensity during the past week (scale 0–10; 0 = no pain, 10 = worst pain possible) and obtain the Oswestry Disability Index (ODI, version 1.0; scale 0–100%: 0% = no disability, 100% = very severe disability). ODI was obtained by a patient-completed questionnaire that generates a subjective percentage score of level of function (disability) in activities of daily living among back pain sufferers [19]. The time interval between baseline MRI and symptom assessment varied from two to six weeks. Standard lumbar spine MRI was performed again at the two-year follow-up visit, 23–25 months after baseline imaging. Questionnaires for average LBP intensity and ODI were completed during the follow-up visit.

### Imaging methods

The MRI studies at baseline were performed with two 1.0 T (Gyroscan Intera, Philips Medical Systems, Eindhoven, The Netherlands) and three 1.5 T (Signa HD, GE Healthcare, Milwaukee, WI, USA and Sonata and Symphony, Siemens Medical, Erlangen, Germany) units using the established spine imaging protocols of the participating hospitals. The imaging parameters of T1- and T2-weighted turbo spin-echo (TSE) or fast spin-echo (FSE) sequences were conventional: for example, 13 ms TE and 600 ms TR (short TE and TR) for T1w and 115 ms TE and 4000 ms TR (long TE and TR) for T2w. At follow-up, all MRI images were obtained with a 1.0 T unit (Gyroscan Intera, Philips Medical Systems), following a uniform protocol [7].

### Image analysis

Evaluations of the baseline and two-year follow-up images were performed by visually examining hard copies of sagittal T1WIs and T2WIs. We chose visual analysis of hard copies for uniformity of assessments, as all participating hospitals did not have digital picture archiving and communication systems (PACS) at the beginning of data collection. All images were assessed by a fellow in musculoskeletal radiology (JJ) who was blinded to the patients’ symptoms. To estimate the interobserver reliability, an experienced musculoskeletal radiologist (JN) evaluated images of 30 endplates from randomly selected patients.

Each patient’s baseline and follow-up MR images were assessed on an x-ray light box, starting with the baseline images. Removal of imaging dates during evaluation of the images was not considered necessary, because the reader was blinded to clinical data. The relative size in percentages (intervals of 5%) of each MC compared to corresponding vertebra in sagittal images was estimated as the average of assessments of three slices (midsagittal and left and right quarter) from T2w. Next, the proportions (%) of M1 and M2 within the MC were estimated from three slices (middle and left and right quarter) covering MC of sagittal T1w and T2w.

### Statistical analysis

Descriptive statistics were calculated to describe the data. Reader reliability was assessed using intraclass correlation coefficients (ICC; absolute agreement). ICC can be interpreted as follows: < 0.40% poor, 0.40-0.59 fair, 0.60-0.74 good, and 0.75-1.00 excellent [20]. Limits of agreement were also calculated. The extent of M1 and M2 at baseline and follow-up were calculated by multiplying the size of MC at both endplates by the corresponding proportions of M1 and M2, respectively, and summing up the products of both endplates. Only M1 and M2 were used in the analyses, due to the low prevalence of M3 (Table 1). Changes in the extent of M1 and M2 over the follow-up were calculated, as well as changes in low back symptoms. Linear regression analysis was used to evaluate the association between changes in the extent of M1 and M2 and low back symptoms, both unadjusted and adjusted for age, gender, and size of MC at baseline. IBM SPSS Statistics version 22 was used in the analyses.

**Table 1:** **Size and proportion of Modic Type 1 (M1), Type 2 (M2) and Type 3 (M3) at baseline and follow-up, and scores of low back symptoms at baseline and follow-up**

	**Baseline**	**Follow-up**
	**(n = 124)** ^**2**^	**(n = 126)** ^**2**^
	**Mean (SD)**	**Mean (SD)**
Size (%)^1^	20.7 (12.3)	24.4 (12.6)
Proportion (%) of		
M1	74.2 (26.2)	40.6 (31.1)
M2	23.9 (26.2)	56.2 (30.2)
M3	1.9 (5.3)	3.2 (8.8)
Low back symptoms		
Low back pain intensity (0–10)	6.5 (1.9)	5.2 (2.7)
Oswestry Disability Index (0-100%)	33.2 (14.4)	28.1 (19.0)

^1^% from vertebral volume.

^2^Endplates with Modic changes.

## Results

### Study population

The baseline study population consisted of 75 chronic LBP patients (87% women) with M1 in the lumbar spine. During follow-up, 11 patients were lost as dropouts: seven due to lack of clinical data and four who were not scanned at follow-up. In all, 64 patients (86% women) were available for the final analyses. Mean age at baseline was 43.8 y (standard deviation [SD] 9.8, range 24–64 y).

### Reliability of image reading

Reliability between the two readers for evaluation of size of MC was excellent (ICC 0.80). Reliability of the evaluation of proportions of M1 and M2 within the observed MC was also excellent (ICC 0.85 and 0.93 for M1 and M2, respectively). Limits of agreement ranged from −10 to 17 (mean difference between JJ and JN 3.4, SD 6.8) for the size of MC, from −44 to 27 (mean −8.8, SD 18.1) for the proportion of M1, and from −20 to 31 (mean 5.6, SD 12.9) for the proportion of M2.

### MRI findings

Most MCs were located at L4/5 or L5/S1 (39% and 49%, respectively, at baseline). The mean size of the MC in relation to vertebral size at baseline was 21% (SD 12, range 5–55%). At the two-year follow-up, the mean size was 24% (SD 13, range 7.5–60%). The mean proportion of the M1 component within the MC was 74% at baseline and 41% at follow-up, while the mean proportion of the M2 component was 24% at baseline and 56% at follow-up (Table 1).

### Clinical symptoms

**Figure 1:**
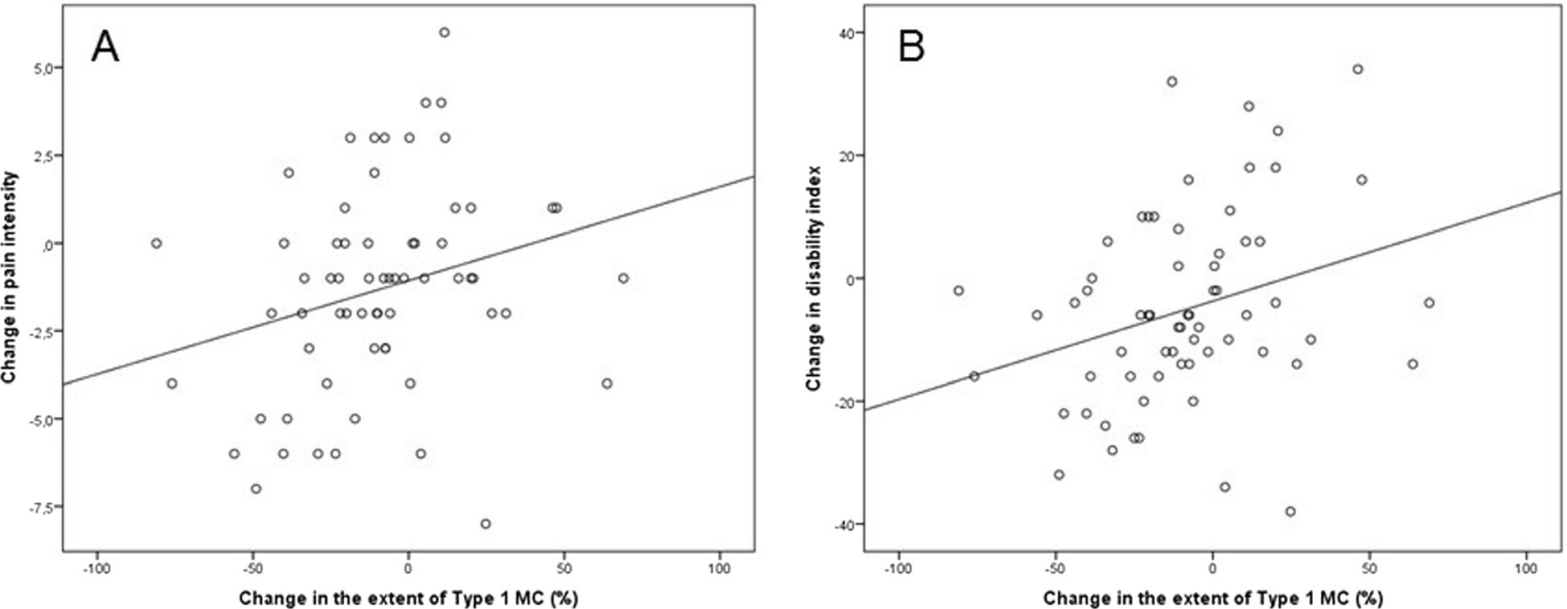
Scatter boxes showing **(A)** the positive correlation between change in the extent of Type 1 Modic change and change in low back pain intensity and **(B)** the positive correlation between change in the extent of Type 1 Modic change and change in Oswestry Disability Index.

**Figure 2:**
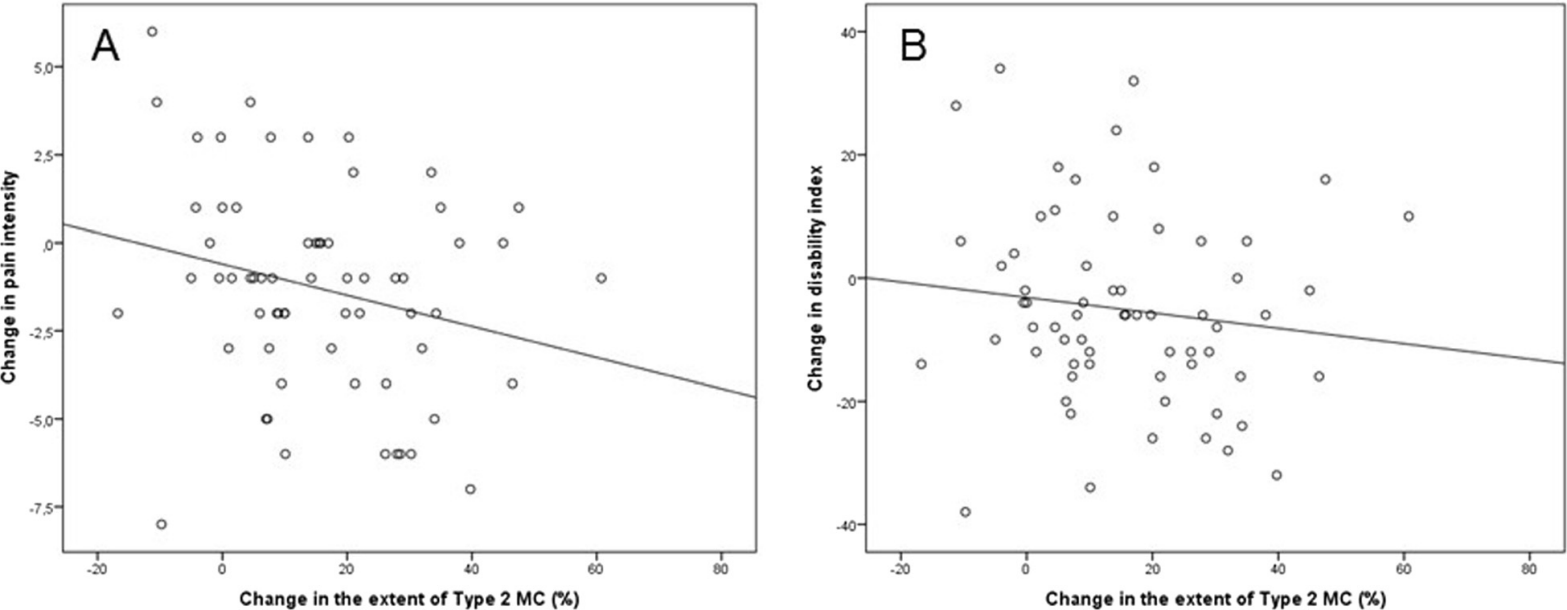
Scatter boxes showing **(A)** the negative correlation between change in the extent of Type 2 Modic change and change in low back pain intensity and **(B)** the negative correlation between change in the extent of Type 2 Modic change and change in Oswestry Disability Index.

At baseline, the mean LBP intensity was 6.5 (SD 1.9, range 1–10) and ODI was 33% (SD 14, range 8–66). At follow-up, the mean LBP intensity was 5.2 (SD 2.7, range 0–9) and ODI was 28% (SD 19, range 0–78; Table 1). Also at follow-up, the intensity of LBP had increased in 15 patients (23%; mean 2.5, SD 1.5) and decreased in 41 patients (64%; mean −3.0, SD 2.0), while the ODI had increased in 19 patients (30%, mean 13.7%, SD 9.8) and decreased in 44 patients (69%, mean −13.4%, SD 9.1) (Figures 1 and 2).

### Association between MRI findings and clinical symptoms

**Figure 3:**
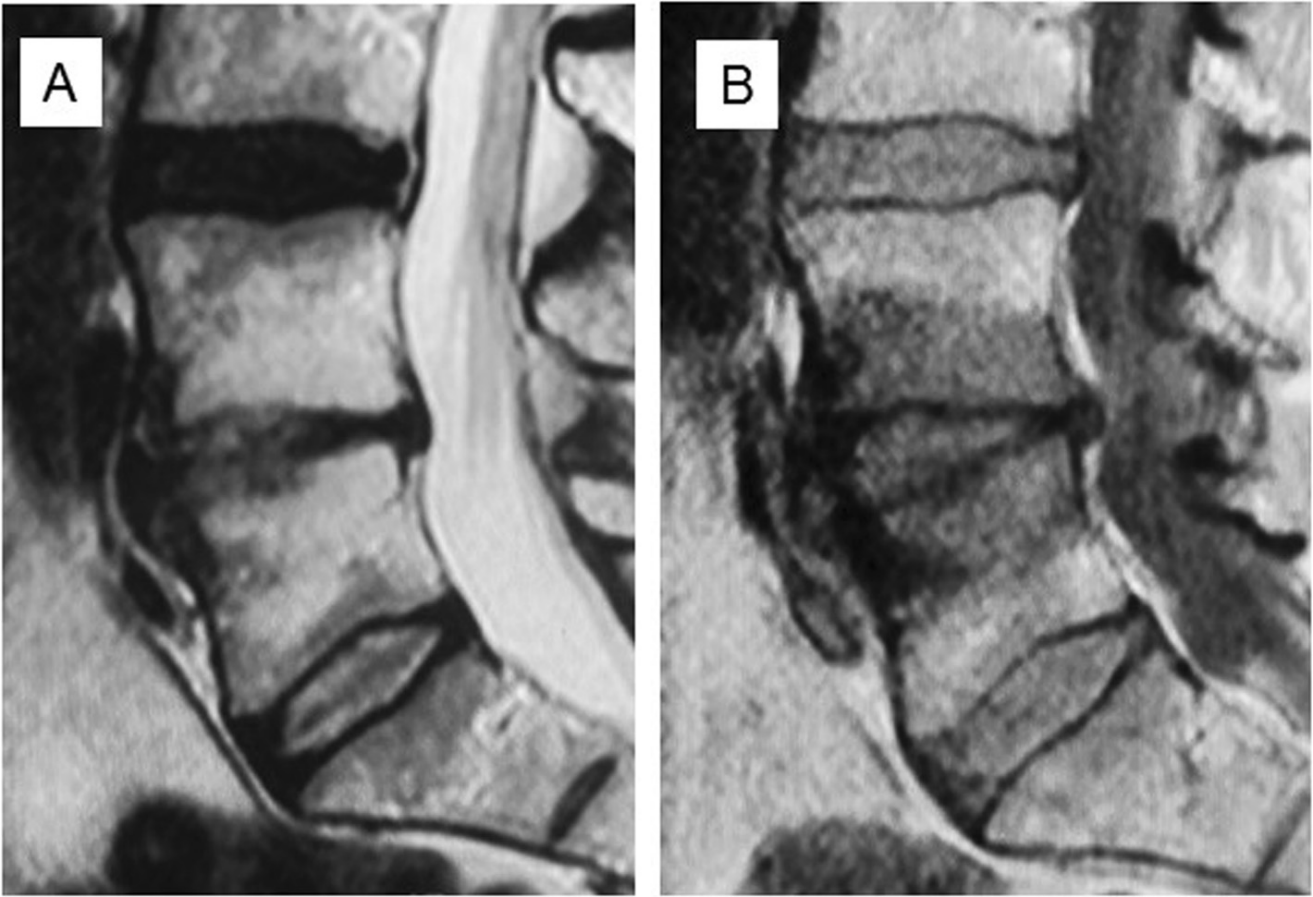
Baseline T2 **(A)** and T1 **(B)** -weighted sagittal MR images of a 45-year-old man with Type 1 Modic change at L4/5. Low back pain intensity 8/10, Oswestry Disability Index 24%.

**Figure 4:**
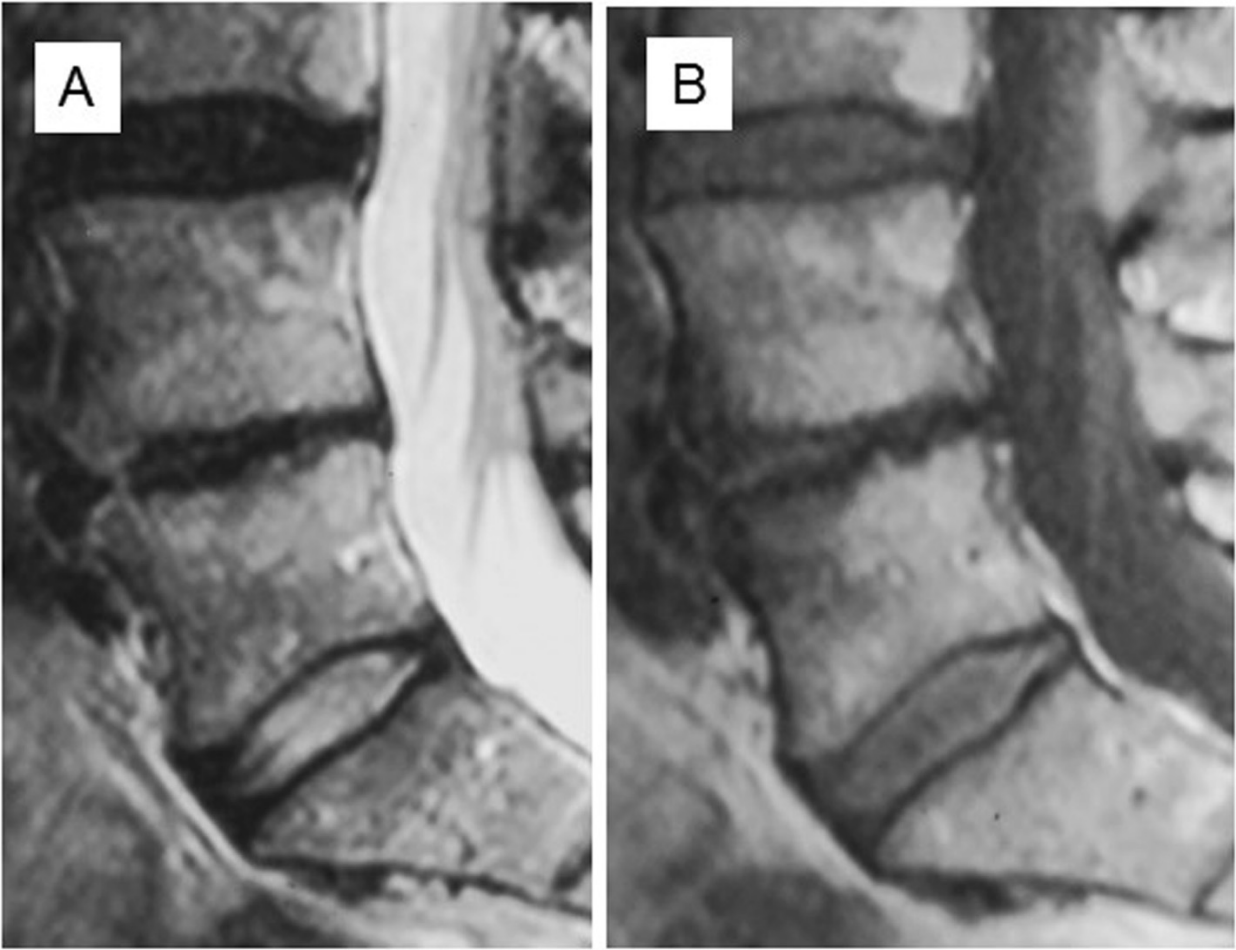
T2 **(A)** and T1 **(B)** -weighted sagittal MR images at follow-up of the same patient as in Figure 1 showing that the Modic change has converted to Type 2. Low back pain intensity 2/10, Oswestry Disability Index 12%.

Change in the extent of M1 associated positively with changes in LBP intensity and ODI (beta 0.26, *p* = 0.036 and beta 0.30, *p* = 0.017; respectively, whereas change in the extent of M2 associated negatively with changes in LBP intensity and ODI (Table 2, Figures 3 and 4). However, this latter association was not statistically significant. When adjusted for age, gender, and size of MC at baseline, the association between the change in the extent of M1 and LBP intensity became non-significant, whereas the association between the change in the extent of M1 and ODI remained significant (Table 2).

**Table 2:** **Association of change in the extent of Modic Type 1 (M1) and Type 2 (M2) with low back pain (LBP) intensity and Oswestry Disability Index (ODI)**

	**LBP intensity**			**ODI**		
	**B (SE)**	**Beta**	**P**	**B (SE)**	**Beta**	**P**
Unadjusted						
Change in M1	0.027 (0.012)	0.26	0.036	0.160 (0.065)	0.30	0.017
Change in M2	−0.044 (0.022)	−0.24	0.054	−0.125 (0.122)	−0.13	0.306
Adjusted for age and gender						
Change in M1	0.025 (0.013)	0.25	0.060	0.166 (0.068)	0.31	0.018
Change in M2	−0.044 (0.023)	−0.24	0.059	−0.128 (0.124)	−0.13	0.304
Adjusted for age, gender and size of Modic change at baseline						
Change in M1	0.019 (0.018)	0.19	0.284	0.282 (0.090)	0.53	0.003
Change in M2	−0.035 (0.024)	−0.19	0.156	−0.135 (0.133)	−0.14	0.315

## Discussion

In this two-year follow-up study, we found significant positive associations between the change in the extent of M1 and changes in both LBP intensity and ODI. The association between changes in M1 and ODI remained significant after adjustment for confounders. Change in the extent of M2 had non-significant negative associations with changes in LBP intensity and ODI. The study sample was chosen to represent patients with M1 in the lumbar spine. The proportion of M1 within MC was 73% on average at baseline. In most patients, the proportion of M1 within MC decreased during follow-up, while the proportion of M2 increased. Both LBP intensity and ODI were more likely to decrease than increase during follow-up. For a 50% decrease in the extent of M1, LBP intensity decreased 2.4 units in VAS, and disability decreased 11.7 units in ODI, while for a 10% decrease the corresponding estimates were −1.3 in VAS and −5.3 in ODI.

It is estimated that <15% of patients who seek care for LBP have symptoms due to a specific cause (e.g., fracture, infection, tumour, or compression of the nerve root). The vast majority of LBP patients are thus classified as having nonspecific LBP. Recently, there has been growing interest in identifying and classifying LBP according to specific clinical subgroups [21]. In some studies, LBP patients with MC have been suggested to be a specific subgroup of LBP [2,22-24]. In a recent prospective cohort study [6], the authors sampled 140 patients to investigate associations between baseline degenerative imaging findings and outcome in sick-listed LBP patients. They concluded that M1 was the only degenerative finding that predicted persistent symptoms and sick leaves. However, the association between MC and LBP remains debatable [25-27].

MC have not only been observed among patients with LBP [4,28], but also in MR images obtained in population-based studies [9,29] and even in asymptomatic subjects [30-32]. Only a few follow-up studies have investigated the associations between low back symptoms and M1 [5,17]. A Danish follow-up cohort study investigated the development of MC over a 14-month period and whether changes in the size and type of MC were associated with changes in clinical symptoms [5]. They concluded that patients with M1 at both baseline and follow-up had a poor prognosis compared to those without M1 at baseline and follow-up. Their results are in accordance with our findings. Moreover, their study design resembles ours, and although our study sample was smaller, we had a longer follow-up. In a longitudinal study by Mitra et al. [17], the authors found a trend of higher pain intensity and disability scores among patients with an increase in the M1 component and lower scores in patients with conversion of M1 to M2.

Our results suggest a statistically significant association between M1 and low back symptoms; even after adjustments, the associations between changes in the extent of M1 and ODI were significant. Hence, our findings support the observation that M1 has a stronger association with LBP than other types of MC [4,16-18,29,33,34]. In a study by Kääpä et al. [18], patients with chronic LBP and M1 suffered from pain and disability significantly more than patients with mixed type M1/2 changes. The authors’ tentative interpretation was that as M1 converts to M2, pain intensity and perceived disability subside. Our finding, which concerned mainly the same study population as that in [18], is in accordance with this interpretation, although a significant association with symptoms was observed only for changes in the extent of M1, not for changes in the extent of M2. In a Finnish study [4], the authors compared self-reported LBP and Modic findings on MRI in a sample of middle-aged male workers. Altogether, 178 MCs in 128 subjects were recorded: 30% M1, 66% M2, and 4% both M1 and M2. They concluded that MC at L5/S1 and M1 lesions were more likely to be associated with pain symptoms than other types of MC, or MC located at other lumbar levels. A study of chronic LBP patients by Kerttula et al. [7] indicated that even during the one-year follow-up, both increasing and decreasing M1 changes were associated with an accelerated process of adjacent disc degeneration, while disc degeneration in the absence of M1 seemed to advance more slowly. M1 may signify a distinct degenerative process in the discovertebral unit [11].

Only two randomized trials have evaluated the efficacy of medication for LBP due to MCs. In a Danish study [35], amoxicillin-clavulanate treatment for three months was effective compared to placebo among patients with M1 after verified disc herniation. In another study [36], zoledronic acid, a long-acting bisphosphonate, was effective in reducing the intensity of LBP in the short-term and in reducing the use of nonsteroidal anti-inflammatory drugs at one-year follow-up among patients with chronic LBP and MC confirmed by MRI. Although these results are promising, more research must be carried out to replicate both treatment interventions.

There are a few limitations that should be considered in our study. First, the study population is quite small. However, solitary M1 or mixed M1/M2 is uncommon, which complicated patient recruitment. Furthermore, we excluded potential clinically relevant changes from other disc levels. Therefore, the patients needed to be collected from several hospitals that used non-identical MR equipment. Differing MR equipment may influence image quality, but uniform imaging protocols were used for high-field MRI. In a Danish study [37], the authors compared lumbar MC in low-field (0.3 T) MRI and high-field (1.5 T) scanners. They concluded that there was a difference between low- and high-field MRI in terms of overall prevalence of MC; the number of MC diagnosed with high-field MRI was significantly higher than with low-field MRI. M1 dominated in low-field scanners, and M2 in high-field scanners. Because differences between the field strengths of our equipment were minor (1.0–1.5 T), we believe that the different scanners should not have a marked influence on the assessment of type or size of MC. The potential influence on LBP symptoms of other degenerative imaging findings (e.g., annular tears and disc bulging), pain medication and other treatments, somatic and psychological comorbidities, educational level, compensation, and other psychosocial elements was not analyzed.

## Conclusions

In this 2-year follow-up study, we found a significant positive association between the change in the extent of M1 and change in ODI. We also found a positive association between the change in the extent of M1 and LBP intensity, which, however, became non-significant after the adjustments. These results lend support to the hypothesis that LBP patients with M1 represent a specific subgroup of patients with LBP.
